# Pharmacological and Toxicological Threshold of Bisammonium Tetrakis 4-(*N*,*N*-Dimethylamino)pyridinium Decavanadate in a Rat Model of Metabolic Syndrome and Insulin Resistance

**DOI:** 10.1155/2018/2151079

**Published:** 2018-06-19

**Authors:** Samuel Treviño, Alfonso Díaz, Eduardo Sánchez-Lara, Víctor Enrique Sarmiento-Ortega, José Ángel Flores-Hernández, Eduardo Brambila, Francisco J. Meléndez, Enrique González-Vergara

**Affiliations:** ^1^Facultad de Ciencias Químicas, Benemérita Universidad Autónoma de Puebla, 14 Sur y Av. San Claudio, Col. San Manuel, 72570 Puebla, PUE, Mexico; ^2^Centro de Química, ICUAP, Benemérita Universidad Autónoma de Puebla, 14 Sur y Av. San Claudio, Col. San Manuel, 72570 Puebla, PUE, Mexico

## Abstract

Vanadium(IV/V) compounds have been studied as possible metallopharmaceutical drugs against diabetes mellitus. However, mechanisms of action and toxicological threshold have been tackled poorly so far. In this paper, our purposes were to evaluate the metabolic activity on dyslipidemia and dysglycemia, insulin signaling in liver and adipose tissue, and toxicology of the title compound. To do so, the previously reported bisammonium tetrakis 4-(*N*,*N*-dimethylamino)pyridinium decavanadate, the formula of which is [DMAPH]_4_(NH_4_)_2_[V_10_O_28_]·8H_2_O (where DMAPH is 4-dimethylaminopyridinium ion), was synthesized, and its dose-response curve on hyperglycemic rats was evaluated. A Long–Evans rat model showing dyslipidemia and dysglycemia with parameters that reproduce metabolic syndrome and severe insulin resistance was generated. Two different dosages, 5 *µ*mol and 10 *µ*mol twice a week of the title compound (equivalent to 2.43 mg·V/kg/day and 4.86 mg·V/kg/day, resp.), were administered intraperitoneal (i.p.) for two months. Then, an improvement on each of the following parameters was observed at a 5 *µ*mol dose: weight reduction, abdominal perimeter, fatty index, body mass index, oral glucose tolerance test, lipid profile, and adipokine and insulin resistance indexes. Nevertheless, when the toxicological profile was evaluated at a 10 *µ*mol dose, it did not show complete improvement, tested by the liver and adipose histology, as well as by insulin receptor phosphorylation and GLUT-4 expression. In conclusion, the title compound administration produces regulation on lipids and carbohydrates, regardless of dose, but the pharmacological and toxicological threshold for cell regulation are suggested to be up to 5 *µ*mol (2.43 mg·V/kg/day) dose twice per week.

## 1. Introduction

Interest in vanadium actions on biological systems has gradually increased, and its physiological relevance has been established in recent years [1, 2]. Vanadium is the 18th most abundant element in the crust of our planet, even more than zinc (0.019% and 0.008%, resp.). Regarding this occurrence, vanadium is present in the soil, water, and air, in almost all types of ecosystems. It is absorbed by plants and travels along the food chain up to humans; thus, it is distributed ubiquitously in living organisms [1–3]. However, metabolism of vanadium in humans and animals has not been fully understood, and evidence is not conclusive as to its importance as a trace element [4]. The estimated daily consumption ranges from 10 to 60 *µ*g depending on a mammal's diet, but its deficiency in mammals inhibits growth, impairs cellular regenerative functions, and affects thyroid metabolism and bone mineralization, and it causes disturbances in lipid and carbohydrate balance [3–6].

Lipid and carbohydrate disorders are strongly linked to obesity development, insulin resistance, type 2 diabetes, dyslipidemia, hepatic steatosis, and cardiovascular diseases. All of which have become known as metabolic syndrome. Metabolic syndrome is a major public health problem and an important clinical challenge worldwide because of its prevalence in 10 to 84% of the population depending on the world region. The International Diabetes Federation estimates that one-quarter of the world's adult population suffers from metabolic syndrome associated with being overweight and an increased body mass index that is reflected in a higher fatty body mass, distributed mainly in the visceral adipose tissue (abdominal circumference) [7–10]. Despite many years of research and partial success in the treatment of metabolic diseases, molecular mechanisms and their accompanying subcellular changes have not been fully elucidated. Therefore, research has focused on less invasive and more effective methods of treatment. Over the last twenty years of research, vanadium compounds have been shown to act in a similar way to insulin on selected diabetes and metabolic syndrome models, in tests both on animals and humans as far as physiological symptoms and biochemical parameters are concerned [11–13].

Depending on its speciation forms, physicochemical properties, and concentration, vanadium can be absorbed and distributed into the body. Most vanadium complexes are transformed into a cationic form (vanadyl) which binds to transferrin or albumin, where it can go through spontaneous oxidation to become vanadate [14]. Vanadate(V) and vanadyl(IV) are easily redox interconvertible, but vanadate is the main inorganic vanadium species available for interaction with cellular functions [2, 15–17]. The oxidation state of vanadium is a factor that determines its biological effects, which are present in the activity regulation of many enzymes and phosphorylation and dephosphorylation processes. In enzymes, regulatory properties of vanadate depend, at least in part, on its esterification with phosphate [18]. Therefore, enzymes can accept vanadate as an analogy to phosphate [19–21]. However, there is a subtle tight threshold between biological activity and toxicity that is correlated to its degree of oxidation (vanadyl < vanadate ion) and chemical form (organic < inorganic) [3, 21–23]. In this respect, the most often observed side effects include a loss of appetite and significant reduction of body weight (often leading to anorexia), as well as a low activity of hepatic damage enzymes, confirmed by histopathological studies [24]. However, reports are limited and divergent, but it has been suggested that vanadium toxicity is dependent on speciation and oxidation state via time and doses of administration. The therapeutic concentration limit of vanadium compounds is, as a general rule, below 0.01 × 10^−3^ M, which is a safe limit and maintains the biological activity, whereas several toxic effects associated with its threshold or accumulation in tissues may be expected to be above 1.0 × 10^−3^ M [25]. Regarding vanadium toxicity, it has been shown that it is dramatically reduced when oligomerized in the form of decavanadate [20–23]. The biochemical importance of decavanadate resides in protein-bound species because it is very stable to pH changes and is eventually turned to labile oxovanadate species. Thus, its rate of disgregation can be very slow but effective, which allows it to exist under physiological conditions for some time [21]. Likewise, decavanadate is an anion that could get into the cell across proteins like channels or lipid interactions to trigger its biological effects on energy transduction systems [26]. Recently, our working team reported a decavanadate compound with DMAPH. The crystal structure of this complex has been completely characterized [27]. Thus, in this study, our aim was to evaluate the possible metabolic activity of the compound in a dyslipidemia and dysglycemia model, related to insulin signaling in liver and adipose tissue, as well as its toxicological and pharmacological effects.

## 2. Materials and Methods

### 2.1. Synthesis of [DMAPH]_4_(NH_4_)_2_[V_10_O_28_]·8H_2_O (V10-DMAP)

All chemicals used were of reagent grade and were purchased from Sigma-Aldrich. The synthesis was performed with a 0.468 g (4 mmol) mixture of ammonium metavanadate in 18 mL of distilled water in an Erlenmeyer flask with magnetic stirring and heated at 70°C until it was dissolved. Then, four drops of concentrated hydrochloric acid (37%) at room temperature were added to allow decavanadate anion formation at pH 6. After obtaining an orange solution from this process, 0.122 g (1 mmol) of 4-dimethylaminopyridine dissolved in 2 mL of distilled water was added dropwise. Once 4-dimethylaminopyridine was added, the mixture was filtered off and allowed to stand at room temperature for one day to produce orange crystals of the title compound [27].

### 2.2. Dose-Response Curve of V10-DMAP

Long–Evans rats were used in this study because this strain is twice as susceptible to spontaneously develop obesity and hyperglycemia. Thirty male rats weighing 300 to 320 g underwent an intraperitoneal application of alloxan (150 mg/kg) to induce hyperglycemia. When hyperglycemia (HG) above 200 mg/dL was present, animals were split into six working groups (*n*=5), all of which were given with dosages of the title compound abbreviated as V10-DMAP from now on, at 0 *µ*mol, 2.5 *µ*mol, 5 *µ*mol, 7.5 *µ*mol, 10 *µ*mol, and 20 *µ*mol, dissolved in 1 mL of injectable solution to be administered via intraperitoneal (i.p.). These dosages were administered twice a week for a month until glucose was normalized (≤130 mg/dL). Monitoring was carried out with serum collected in a BD Vacutainer® venous blood collection system, centrifuged at 2500 rpm for 5 min. Glucose quantification was carried out with a commercial kit, and the values obtained were used to determine a dose-response curve.

### 2.3. Dyslipidemia and Dysglycemia Model

Forty male Long–Evans rats (70–100 g) were provided by the vivarium “Claude Bernard” from Benemérita Universidad Autónoma de Puebla. The rats were housed in a climate-controlled environment and 12 h light-dark cycles with free access to food and water *ad libitum*. All procedures described in this study agreed with the Guide for the Care and Use of Laboratory Animals of the Mexican Council for Animal Care NOM-062-ZOO-1999. All applicable international, national, and institutional guidelines for the care and use of animals were followed. The rats were conditioned with a normal calorie diet during 15 days. The diet used was 5001 from LabDiet (Laboratory Rodent Diet), and its composition can be accessed on the manufacturer's website. Upon reaching the ideal weight (150 g), animals were randomly split into different groups. Two groups were formed: group 1: normal calorie or NC group fed with 5001 from LabDiet (*n*=10); group 2: high calorie or HC group fed with 5008 from LabDiet (*n*=30) Diet 5001 from LabDiet provides 58.0% carbohydrates, 13.5% fat, and 28.5% proteins. Diet 5008 from LabDiet provides 56.44% carbohydrates, 17.0% fat, and 26.85% proteins. The groups were fed for two months *ad libitum* with diet 5001 as a caloric control and diet 5008 until they developed the dyslipidemia and dysglycemia model. The model was validated by measuring body weight, abdominal perimeter, and tip of the nose to base of the tail length. BMI (body mass index) and body fat percentage were determined by the Lee index [28]. Glucose, fructosamine, triglycerides, total cholesterol, HDL cholesterol, insulin, and insulin resistance by a homeostasis model assessment insulin resistance (HOMA-IR) and insulin resistance adipocyte dysfunction (IDA-IR) were biochemically evaluated [28].

### 2.4. Effect of V10-DMAP on Dyslipidemia and Dysglycemia Model

After appearance of the metabolic dysregulation validation, two groups of 10 rats from the 5008 group were randomly separated and divided into groups 3 and 4 as follows: group 3: HC-V10-DMAP-5 *μ*mol group fed with diet 5008 and administered twice a week (i.p.) (*n*=10); group 4: HC-V10-DMAP-10 *μ*mol group was fed with diet 5008 and administered twice a week (i.p.) (*n*=10). Both groups were fed for a period of two more months. The zoometry was monitored every two weeks for two months as described above. Toxicological and biochemical parameters in fasting conditions were assessed in serum at the end of the experimental period: total bilirubin, aspartate aminotransferase (AST), alanine aminotransferase (ALT), glutamyl transpeptidase (GT), lactate dehydrogenase (LDH), glucose, insulin, leptin, adiponectin, fructosamine, triglycerides, free fatty acids, total cholesterol, and HDL cholesterol were determined by using commercial kits. Additionally, an oral glucose tolerance test (OGTT, 1.75 g of glucose anhydrous/kg) was carried out after 4–6 h of fasting. A postload monitoring was carried out at 30, 60, and 90 min, and the area under the curve (AUC) was calculated. The samples were taken by cardiac puncture after anesthesia with ketamine and xylazine (20/137 mg/kg). Insulin resistance indexes (HOMA-IR and IDA-IR) were calculated.

### 2.5. Effects of V10-DMAP on the Liver and Adipose Tissue

One week after the last biochemical evaluation, rats were anesthetized with sodium pentobarbital (40 mg/kg, i.p.) and then perfused with 200 mL of 4% paraformaldehyde. Biopsies from liver and visceral white adipose tissue were removed and postfixed in the same solution for 48 h and then embedded in paraffin. Sections of 5 *μ*m thick were taken from each tissue for subsequent staining. Histological evaluation was carried out with hematoxylin-eosin stain using standard procedures after paraffin removal and tissue rehydration. The degree of hepatic injury and adipose tissue remodeling (hypertrophy) were evaluated with light microscopy. Additionally, sections of tissue were rehydrated according to conventional histological techniques; then, nonspecific binding sites were blocked by incubation in 2% IgG-free bovine serum albumin (BSA, Sigma). After that, specimens were incubated with 0.2% Triton X-100. The sections were incubated overnight at 4 to 8°C with primary antibodies: *p*-insulin R*β* Antibody (Tyr 1162/1163) to the liver and adipose tissue and GLUT-4 to adipose tissue. The primary antibodies (Santa Cruz Biotechnology Inc., CA, USA) were 1 : 100 diluted. Fluorescein isothiocyanate (FITC) secondary antibodies (1 : 100, Jackson ImmunoResearch Laboratories Inc., PA, USA) revealed absence or presence of proteins. Slides were mounted with VectaShield containing 4′,6-diamidino-2-phenylindole (DAPI) (Vector Labs., CA, USA) for nucleus staining. Photomicrographs were taken using a fluorescence microscope (Leica Microsystems GmbH, Wetzlar, Germany) and projected with a Leica IM1000 version 1.20 release-9 computer-based program (Imagic Bildverarbeitung AG, Leica Microsystems, Heerbrugg, Switzerland).

### 2.6. Statistical Analysis

Results were expressed as a mean ± standard error of the mean (SEM). A dietary comparison was performed by Student's *t*-test, considered significant *p* ≤ 0.05. Meanwhile, a comparison of the groups studied after (V10-DMAP) treatments was performed by an analysis of variance and multiple comparisons using a two-way ANOVA analysis and a Bonferroni post hoc test, obtaining a significant *p* < 0.05. A GraphPad Prism 5.0 statistical program was used to perform this analysis.

## 3. Results

**Figure 1:**
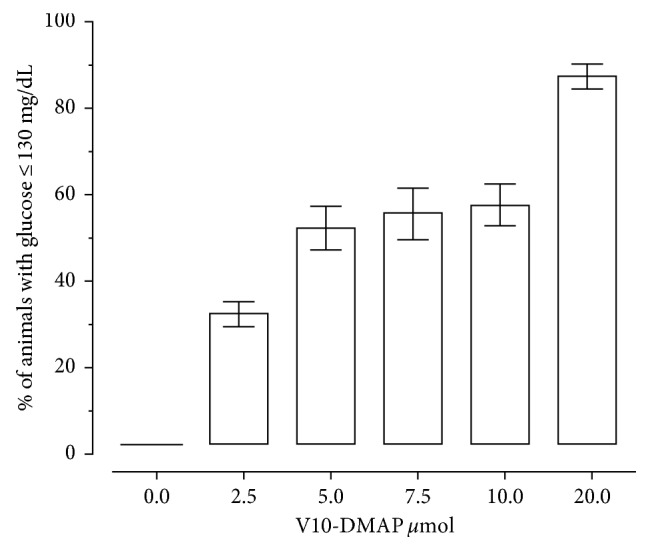
Effective dose 50 analysis (ED_50_). The figure represents the percentage of glucose level reduction ≤130 mg/dL by administration of V10-DMAP. The hyperglycemic Long–Evans rats (*n*=5/group) were given with dosages of 0.0, 2.5, 5.0, 7.5, 10.0, and 20 *µ*mol of V10-DMAP for four weeks. The Long–Evans rats were alloxan-induced hyperglycemic (150 mg/kg). Mean glucose before V10-DMAP administration was ≈300 mg/dL.

Hyperglycemic rats induced by alloxan initially showed an average of serum glucose of 300 mg/dL. The V10-DMAP dosages administrated twice a week for one month period produced a decrease in glucose levels depending on the vanadium compound concentration. According to the results, doses of 5 *µ*mol and 10 *µ*mol of the title compound (equivalent to 2.43 mg·V/kg/day and 4.86 mg·V/kg/day, resp.) twice a week, reduced glucose levels below 130 mg/dL in 50% of the subjects given with this compound (Figure 1). To determine the effective dose 50 (ED_50_), we proceeded to perform a mathematical model to apply the exact concentration. Because the mathematical model did not discriminate between 5, 7.5, and 10 *μ*mol doses, we decided to use only two different doses (5 *µ*mol and 10 *µ*mol, equivalent to 2.43 mg·V/kg/day and 4.86 mg·V/kg/day, resp.) in later experiments of metabolic regulation to investigate their toxicological effects.

On the contrary, in the model of dyslipidemia and dysglycemia produced by one-month consumption of diet 5008 (which possesses 3.5% more fat than the normal-caloric diet 5001), there was a Long–Evans rat weight increase. Also, there was an elevation in the levels of glucose, fructosamine (protein glycation), and insulin levels with hepatic insulin resistance (HOMA-IR) and adipose resistance (IDA-IR) (Table 1). Moreover, hypertriglyceridemia and hypoalphalipoproteinemia (low level of HDL) were observed. These results strongly suggest the development of metabolic syndrome; however, the Long–Evans rats were fed with 5008 diet for one more month to confirm the model. After the second month, the Long–Evans rats showed a 20% weight gain in comparison to the diet 5001 group (Table 1). Similarly, glucose increased twice as much (206 mg/dL), fructosamine increased 40%, and insulin increased by 118%, which produced a noticeable insulin resistance level both in the liver and adipose tissue. Dyslipidemia showed a 71% triglyceride increase and a 15% HDL decrease (Table 1). These two-month-period results ensure the development of the dysglycemia and dyslipidemia model. Then, rats suffering from metabolic disorder were separated in individual cages, and V10-DMAP dosages of 5 *µ*mol and 10 *µ*mol (equivalent to 2.43 mg·V/kg/day and 4.86 mg·V/kg/day, resp.), twice per week, were administered intraperitoneally. Diet consumption was maintained *ad libitum*.

**Figure 2:**
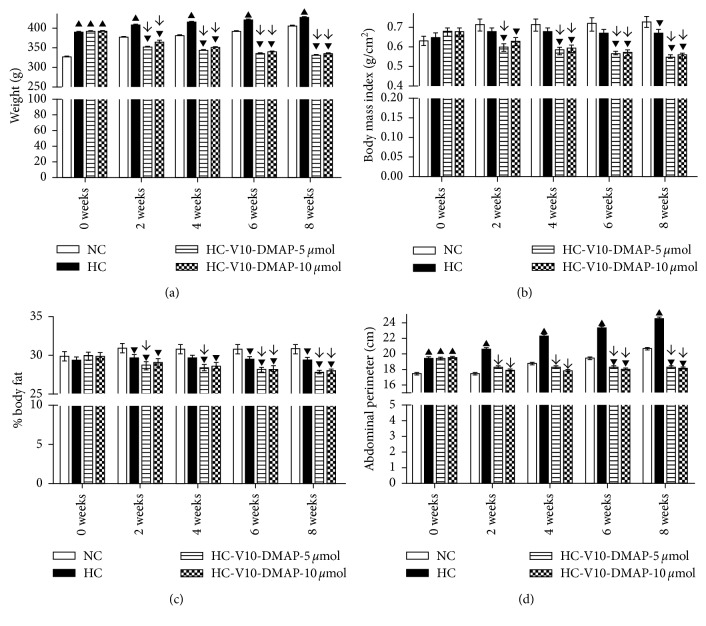
Zoometry after diets and V10-DMAP treatments: (a) body weight, (b) BMI, (c) Lee index, the percentage of the body fat mass index, and (d) abdominal perimeter or circumference. Results shown are the average ± SEM. ▲ indicates a significant difference between values above the control group with a normal calorie diet. ▼ indicates a significant difference between values below the control group with a normal calorie diet. ↓ indicates a significant difference between values below the HC group. Comparisons between groups were performed by two-way ANOVA and the Bonferroni post hoc test; *p* < 0.05.

To justify the fact that the V10-DMAP dose may be used in the treatment of metabolic diseases, both zoometry and biochemical parameters were evaluated in all groups. Overweight was observed in groups which fed a HC diet (time dependent). Rats starting with an approximate 390 g weight showed a 36 g weight gain (426 g 8 weeks later). These results mean 5.5% more weight than the control group (Figure 2(a)). Nonetheless, BMI showed no change, and fat mass percentage showed a significant decrease in the HC diet group. Remarkably, fat mass increased in the abdominal circumference area, suggesting impaired visceral white adipose tissue, because there was an increase of 8% in 8 weeks (Figure 2(d)).

**Figure 3:**
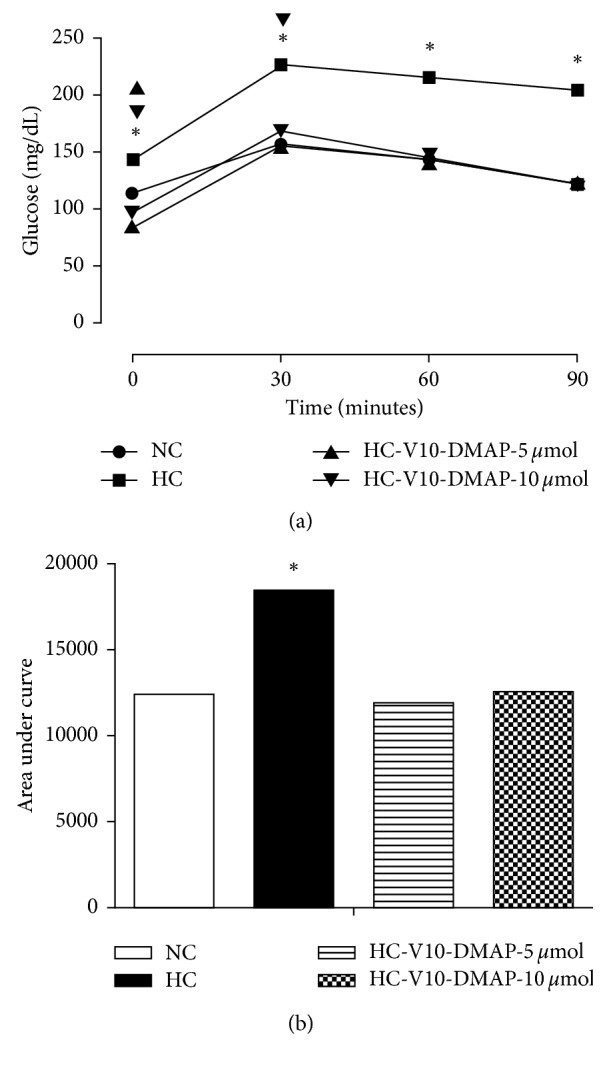
Oral glucose tolerance test and area under the curve after V10-DMAP administration: (a) OGTT (1.75 g/kg weight of anhydrous glucose) (*n*=10 per group) and (b) graphical representation of AUC analysis. Results shown are the average ± SEM. ^*∗*^, ▲, and ▼ indicate a significant difference among the HC, HC-V10-DMAP-5 *μ*M, and HC-V10-DMAP-10 *μ*M groups, respectively, in comparison to the NC group. Comparisons between groups were performed by two-way ANOVA and Bonferroni's post hoc test; *p* < 0.05.

Weight was reduced in both groups of rats given with V10-DMAP since the second week was in relation to the NC group. At the end of the V10-DMAP administration, group 3 (5 *µ*mol) and group 4 (10 *µ*mol) showed a significant weight reduction of 18% in relation to the NC group, while both were 22.5% lower about the HC group (Figure 2(a)). Likewise, the BMI of groups 3 and 4 improved after two weeks of V10-DMAP administration in 16.5% and 12%, diminishing up to 24.5% after eight weeks in comparison to the NC group and 17.5% in comparison to the HC group (Figure 2(b)). The body fat percentage had a similar pattern. The body fat percentage after two weeks had a similar pattern since the second week in groups 3 and 4, decreasing 7% and 6%, respectively. At the end of the experiment (8 weeks), the fat percentage was 10% lower in the NC group and 5% lower in the HC group (Figure 2(c)). Finally, abdominal perimeter showed a clear reduction. The maximum difference was 11% after eight weeks in groups 3 and 4 in comparison to the NC group, whereas the difference was 25.5% in comparison to the HC group for both groups (Figure 2(d)). Similarly, the oral glucose tolerance test showed significant differences in fasting (0 min) between the V10-DMAP groups in comparison to the NC group, but at 30 min, the V10-DMAP-10 *µ*mol group was also slightly higher. Nevertheless, the area under curve (AUC) analysis showed no difference in both V10-DMAP groups. The HC group was evidently different in comparison to the NC group with a 26.5% increase at 0 min, a 48% increase at 30 min, a 45.5% increase at 60 min, a 69% increase at 90 min, and a 48.5% at AUC (Figures 3(a) and 3(b)).

Complete biochemical effects are shown in Table 2. In comparison to the NC group, the high-fat diet in the HC group maintains metabolic dysregulation. Meanwhile, the V10-DMAP administration shows substantial improvements in biochemical parameters. Both groups 3 and 4 regulate their fasting glucose and fructosamine levels, as well as insulin secretion until similar levels are reached as in the NC group. Therefore, hepatic and adipose insulin resistances disappear, and adipokine secretion was almost regulated. In the HC-V10-DMAP-10 *µ*mol group, leptin remained slightly high, whereas adiponectin improved, but it did not recover the level shown by the NC group. In this regard, the HC-V10-DMAP-5 *µ*mol group showed an adipokine and a lipid profile return to normal levels, but triglycerides did not diminish like the NC group. Complete lipid regulation was observed in the HC-V10-DMAP-10 *µ*mol group. Notably, HDL level in both groups given with V10-DMAP showed an increase in its concentration, even higher than the NC group. All biochemical parameters in the V10-DMAP-administered groups presented an improvement in comparison to the HC group.

On the contrary, biochemical parameters related to hepatic toxicological effects were measured (Table 3). Total bilirubin showed no differences in either group. However, in both the HC group and group 4, the hepatic damage enzymes are increased to 65% and 26.5% in AST, increased to 38.5% in ALT (only in the HC group), 38% and 27% in *γ*-GT, 27% and 58% in LDH, respectively. Group 3, which belongs to HC-V10-DMAP-5 *µ*mol, showed no differences in comparison to the NC group, and all serum biomarkers showed significant differences in comparison to the HC group.

**Figure 4:**
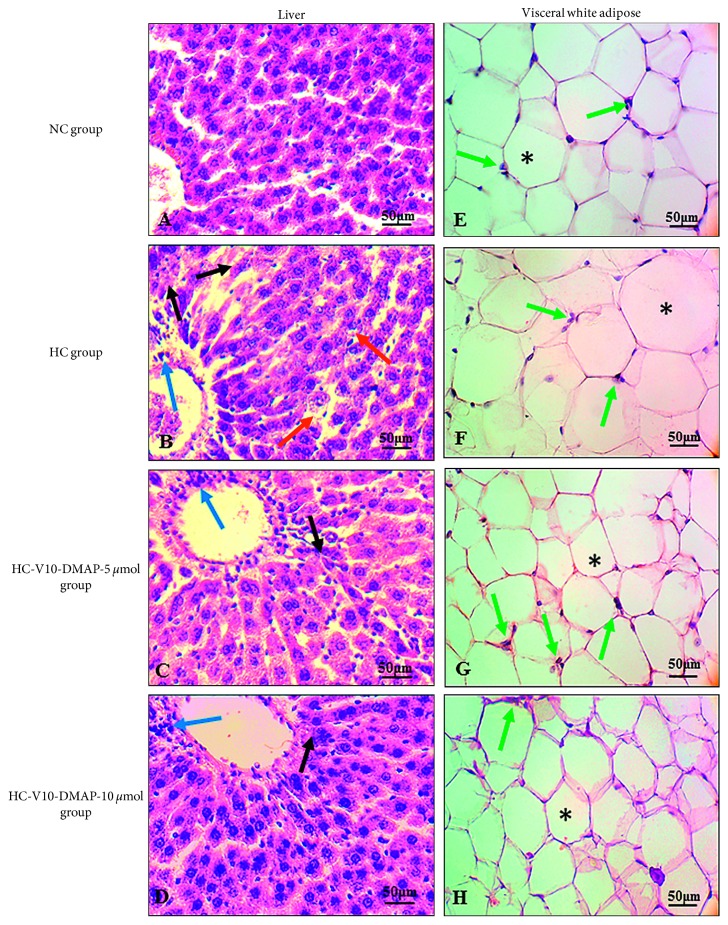
Histological features in the liver and visceral white adipose tissue after V10-DMAP administration. (a)–(d) Liver biopsies with H&E staining. The central vein was taken as a reference. (e)–(h) Visceral adipose tissue biopsies with H&E staining. Magnification images were acquired with a 40x objective. The blue arrow indicates inflammation by infiltrating leukocytes. The black arrow indicates hepatic cell necrosis. The red arrow indicates balloon cell or Mallory–Denk bodies. The asterisk indicates triglyceride storage area in adipocytes. The green arrow indicates blood vessels.

Histological changes were evaluated in tissue sections stained with hematoxylin and eosin (H&E). These changes were viewed under standard bright field illumination. Eosinophilic cytoplasm and blue nuclei, which are characteristic of liver cells, were observed in the liver of the NC group with a distribution of the sinusoid spaces taking the central vein as a reference (Figure 4). The HC group showed visible histology changes, including structure damage, hepatocellular necrosis (black arrow), leukocyte infiltration, recognized as portal inflammation (blue arrow), necrosis, and development of Mallory–Denk bodies, which are an inclusion found in the cytoplasm of hepatocytes. Also, sinusoids between the hepatocyte plates were noticeably enlarged in the liver (Figure 4). Despite that, injuries were of low grade. Liver injury notably diminishes with 5 and 10 *µ*mol doses of V10-DMAP, but weak portal inflammation (blue arrow) and slight focal confluent necrosis were observed (Figures 4 and 4).

On the contrary, normal histology of adipose tissue is shown in Figure 4. Regarding this type of histology, loose connective tissue composed mostly of adipocytes and their storage space (asterisk), and blood vessels can be observed (green arrow). Meanwhile, the HC group showed adipocyte hypertrophy which is characterized by fewer adipocytes (number per analyzed area) with a vascularization increase (Figure 4). Conversely, adipocytes in both groups V10-DMAP reduced their storage area, increasing cell number and vascularization per analyzed area (Figures 4 and 4).

**Figure 5:**
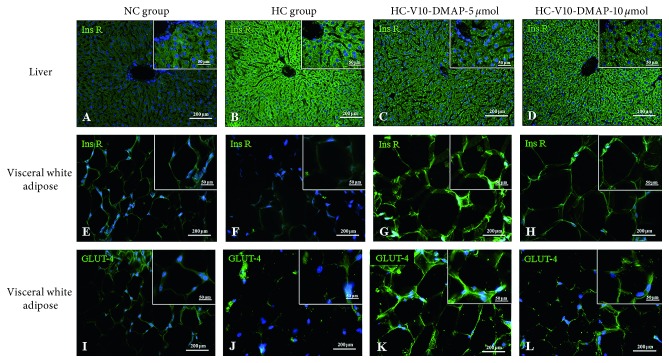
Insulin receptor and GLUT-4 in the liver and visceral white adipose tissue after V10-DMAP treatments. Photomicrographs show immunoreactivity to the insulin receptor and GLUT-4, both marked in green and counterstain with DAPI for nuclei, in the liver and adipose tissue. (a)–(d) Hepatic biopsies, insulin receptor phosphorylated in tyrosine (Tyr 1162/1163), and magnification 20x. (e)–(h) Visceral white adipose biopsies, insulin receptor phosphorylated in tyrosine (Tyr 1162/1163), and magnification 40x. (i)–(l) Visceral white adipose biopsies, glucose transporter type 4 insulin-dependent, and magnification 40x.

To understand the effects caused by the administration of the V10-DMAP compound, the immunoreactivity in green for the insulin receptor phosphorylated in tyrosine (Tyr 1162/1163) was analyzed in the four study groups. Arbitrary units (pixels) were determined and normalized to the NC group using ImageJ program (data not shown). Regarding the control group, immunoreactivity to hepatic insulin receptor was higher in the HC group (120%), HC-V10-DMAP-5 *µ*mol group (56%), and HC-V10-DMAP-10 *µ*mol group (81%) (Figures 5–5). In visceral white adipose tissue, immunoreactivity in the HC group was 6% less than the NC group, while there was a 104% increase in the HC-V10-DMAP-5 *µ*mol group but only a 20% increase in the HC-V10-DMAP-10 *µ*mol group (Figures 5–5). Finally, GLUT-4 immunoreactivity in the HC group and HC-V10-DMAP-10 *µ*mol group was a 54% and 28% less than the NC group, but there was some improvement in comparison to the HC group. Meanwhile, immunoreactivity for GLUT-4 in the HC-V10-DMAP-5 *µ*mol group was higher (33%) to the NC group (Figures 5–5).

## 4. Discussion

In this paper, we evaluated the metabolic activity of V10-DMAP on the dyslipidemia and dysglycemia model. Previously, our working team reported the synthesis and characterization of V10-DMAP by infrared spectroscopy, thermal analysis, and single crystal X-ray diffraction [27]. Similarly, we have previously synthesized and reported another hybrid material based on metformin, a popular drug used to treat type 2 diabetes mellitus [29]. Metformin decavanadate has shown hypoglycemic and hypolipidemic effects on models of type 1 and type 2 diabetes mellitus. Despite vanadium compounds having insulin-mimetic properties, and the first report on their therapeutic properties about diabetes and dyslipidemia appeared in the 20th century [30, 31], its mode of action is still poorly understood at the molecular level [30, 32–39]. Although the general population is exposed to vanadium, normal blood concentration rarely exceeds 0.2 *µ*M [3, 18, 40]; nonetheless, the therapeutic dose is controversial, because patients have a dependence of vanadium speciation and dose level.

In this regard, results showed that a micromole dose of decavanadate is sufficient to reach a hypoglycemic effect. Doses of 5 and 10 *µ*mol (equivalent to 2.43 mg·V/kg/day and 4.86 mg·V/kg/day, resp.) showed no difference to discriminate between them. However, when toxicological effects were analyzed, the 10 *µ*mol dose had a poor effect on AST, ALT, and LDH activities and did not improve γ-GT activity in comparison to the 5 *µ*mol dose. Toxicological effects of decavanadate, usually in excess, are associated with the production of reactive oxygen species, lipid peroxidation, Fenton reaction increase, and mitochondrial antioxidant enzymes decrease, such as superoxide dismutase (SOD) and catalase activities, which lead to cell death and, releasing transaminases to plasma [1, 2, 22, 23, 41, 42]. Additionally, decavanadate in the range of 1 to 10 *µ*mol produced 50% loss of cell viability in cardiomyocyte cultures [43]. Necrosis is involved in cell death by decavanadate excess, which is shown in Figure 4 where necrosis and inflammation were observed by infiltrating leukocytes, mainly in the V10-DMAP-10 *µ*mol group, though there was a reduction of balloon cells or Mallory–Denk bodies and fatty deposits. Meanwhile, the 5 *µ*mol (2.43 mg·V/kg/day) dose regulated the enzymatic activities and improved liver histology. In rodents, dosages that normalize glycemia were between 60 and 100 mg·V/kg/day, which produced vanadium levels in blood ranging from 10 to 20 *µ*M. In humans, a dosage of 1.5 mg·V/kg/day has reported control of glycemia. On this condition, vanadium in blood is 1 to 5 *µ*M [3]. Although in other studies the insulin-mimetic effects of multiple vanadium species (vanadate, peroxovanadates, vanadyl sulfate, etc.) and their powerful hypoglycemic effect have been reported, the ED_50_ and toxic effects or damage to tissues by vanadium administration have not been shown. Our data suggest that vanadium concentration and the species administered are very important not only for glucose homeostasis but also for the toxicological threshold. Therefore, micromole quantities of decavanadate seem to be a highly druggable polyoxovanadate form.

Thus, it is possible to consider a new prodrug concept that implies dissociation of vanadium complexes before they reach the target biomolecules [44]. In this regard, decavanadate species are usually not considered in vanadium toxicological studies, mainly by speciation of vanadyl (V^IV^O)^2+^ or vanadate (V^V^O_4_)^3−^ [45]. It has been proposed that the decomposition rate of decavanadate is slow and can promote either vanadyls or vanadates depending on the cell environment and its requirements, so in the body, its half-life expands hours (few hours in plasma, close to 26 hours in tissues, and after 10 days in urine) [18]. Therefore, the pharmacological activities can be exerted for long periods with a smaller dose that does not reach the toxicological threshold [46, 47]. During the time that it remains in the body, decavanadate can exert the biological actions.

The Long–Evans rats fed with lipid-rich diet 5008 developed a severe metabolic disorder in lipid and carbohydrates, as well as insulin resistance and hyperleptinemia with hypoadiponectinemia (Table 2 and Figure 3). Additionally, the Long–Evans rats observed overweight and visceral obesity (Figure 2). In adipocytes, hypertrophy and hyperplasia were evident with an increased area of stored triglycerides and impaired vascularization (Figure 4). Likewise, a decrease in phosphorylation of insulin receptor without GLUT-4 immunoreactivity was observed (Figures 5 and 5). Meanwhile, hepatic tissue (Figure 4) showed necrosis, fibrosis, inflammation, fatty droplets, Mallory–Denk bodies, and an increase in phosphorylation of insulin receptor (Figure 5). These results are completely associated with insulin resistance, dyslipidemia, and metabolic syndrome. Some studies based on hyperinsulinemic-euglycemic clamp experiments have consistently demonstrated a whole-body insulin resistance in animal fat-fed rodents [48], thus matching with the biochemical parameters and the excess caloric intake reported herein. Insulin resistance is usually accompanied by hyperinsulinemia that promotes an ectopic lipid accumulation and a glucose transport decrease [49, 50]. Ectopic lipid accumulation is recognized as steatosis that leads to lipotoxicity and is strongly linked to insulin resistance, metabolic syndrome, and diabetes mellitus development [51, 52].

Particularly, fat mass accumulation during obesity development is characterized by adipocyte hyperplasia and hypertrophy, which increases the production of proinflammatory adipokines that lead to a state of chronic low-grade inflammation and may promote obesity linked to metabolic disorders, such as insulin resistance and metabolic syndrome [7]. Inflammation may impair insulin action systemically by increasing free fatty acids (FFA) or proinflammatory adipokines, such as leptin, which is a key hormone involved in the regulation of satiety, energy intake, and energy expenditure. Thus, hyperleptinemia does not suppress appetite, a phenomenon known as leptin resistance [53–57]. Leptin resistance is thought to be a fundamental pathology in nonalcoholic fatty liver development, insulin resistance, glucose tolerance impairment, and dyslipidemia as was observed in rats fed with diet 5008. On the contrary, adiponectin regulates lipid and glucose metabolism and increases insulin sensitivity, regulates food intake and body weight, and protects against chronic inflammation. It also increases glucose transport in muscles and enhances fatty acid oxidation, so it is considered anti-inflammatory, antiatherogenic, anti-insulin resistance, and antimetabolic syndrome adipokine [7, 58–62]. Metabolically dysregulated rats showed severe hypoadiponectinemia and consequently insulin resistance in liver and adipose tissue. Insulin resistance implies changes upon insulin signaling, mainly in a transphosphorylation reaction that activates the insulin receptor substrate (IRS), an intrinsic kinase that activates the phosphatidylinositol 3-kinase- (PI3K-) AKT/protein kinase B (PKB) pathway to modulate most metabolic functions of insulin, such as glucose transport by GLUT-4 in adipocytes and myocytes. Meanwhile, this pathway modulates glycogen synthesis, gluconeogenesis, and protein synthesis in liver [63]. On the contrary, it also signals cell growth by the Ras-mitogen-activated protein kinase (MAPK) pathway [63–65]. Inflammatory adipokines enhance degradation of IRS1/2 by the protein tyrosine phosphatase 1B (PTP1B) activity. Therefore, GLUT-4 protein diminished in adipocytes, impairing glucose tolerance response in return to basal levels after a glucose load. In addition, it promoted triglyceride synthesis in the liver associated with insulin receptor hyperphosphorylation, as observed in rats which were fed a high-lipid diet.

It is remarkable that, since its early discovery, the insulin-like activity of vanadium has been considered to be an insulin-mimetic agent that reaches glucose regulation by similar pathways to the hormone. However, the metabolic model, where the defect is caused by hyperinsulinemia and insulin resistance, has not considered that the hormone is at high levels. Therefore, if vanadium has only affected, such as an insulin-mimetic agent, the defect would be promoted. In this respect, vanadium has proven to be a powerful inhibitor of protein tyrosine phosphatases (PTPs), improving tyrosine phosphorylation of IRS1/2 in rat adipocytes and cardiomyocytes, which lead to activation of its associated PI3K activity [66, 67] and PKB [68–70], stimulation of glucose uptake [71, 72], and GLUT-4 translocation [73, 74]. Also, vanadium in liver enhances phosphorylation of ERK1/2, PKB, and glycogen synthase kinase- (GSK-) 3*β* [71, 75]. As a result, vanadium acts efficiently on insulin phosphorylation cascade, probably because of its structural analogy between vanadate and phosphate, where the monomeric vanadate is slightly larger than the phosphate [18]. Moreover, several studies suggest that vanadium is involved in the regulation of phosphate-dependent processes, such as metabolic processes involving phosphatases and kinases. However, the toxicological threshold of vanadium compounds must always be taken into consideration because more metal into cells may cause damage or simply a loss of efficiency. As it can be seen, the V10-DMAP-10 *µ*mol group does not reach the insulin receptor phosphorylation levels in both adipose tissue and liver compared with the V10-DMAP-5 *µ*mol group, where even GLUT-4 protein is regulated more efficiently.

Although the vanadium mechanism on metabolic regulation has been described, decavanadate as an insulin-mimetic agent or enhancer is not fully understood, but its insulin-mimetic effects are probably related to the inhibition of tyrosine phosphatase-1B (PTP1B) [45]. Recently, it has been reported that the signal transduction pathway of PTP1B is influenced by reactive oxygen species (ROS) generated into metabolic cascades [44], so vanadium may be able to act as insulin mimetics due to changes in prooxidant and oxidant balance. In this respect, decavanadate has been observed to inhibit mitochondrial respiration of hepatocytes with 100-fold more power than vanadate, diminishing oxygen consumption and ROS production, which is related to mitochondrial complex III [43]. Inhibition of mitochondrial complexes make cells require more energy, so the burning of lipids and carbohydrates and the FFA flux from adipocytes to hepatocytes are continuously maintained for the adipose tissue to return to a morphology similar to the control group. Thus, the adiponectin level was regulated (decreasing leptin and increasing adiponectin). Meanwhile, decreased fatty content and glucose balance in the liver are improved, so glucose tolerance is also improved, and there are less fructosamine formation and insulin need, leading to insulin resistance reduction, both in adipocytes and hepatic tissue. Therefore, hepatocytes showed an injury reduction. Notably, the 5 *µ*mol (equivalent to 2.43 mg·V/kg/day) dose was more efficient than the 10 *µ*mol (4.86 mg·V/kg/day) dose. Supporting these facts, in previous studies, our working team has demonstrated that metforminium decavanadate produces metabolic regulation on carbohydrates and lipids in hyperinsulinemia and hypoinsulinemia models [76–79], regardless of the insulin-mimetic action by vanadium.

## 5. Conclusion

The V10-DMAP administration, only twice per week, produced regulation on lipids and carbohydrates, regardless of dose administered here; yet, the V10-DMAP-5 *µ*mol (2.43 mg·V/kg/day) dose showed more effective effects than the V10-DMAP-10 *µ*mol (4.86 mg·V/kg/day) dose in the liver, which was confirmed by enzymatic activity. In adipose tissue, the V10-DMAP-5 *µ*mol (2.43 mg·V/kg/day) dose was also more beneficial on cells and adipokines. In this regard, a low decavanadate dose produced a clear regulation of the insulin signaling pathway. Furthermore, different experiments strongly suggest that vanadium and its species in the cell may act on proteins, such as phosphorylases and kinases, so it should not be considered only as an insulin-mimetic agent, and a deeper understanding of its mechanism of action needs to be further investigated. The pharmacological and toxicological threshold for cell regulation are suggested to be up to 5 *µ*mol (2.43 mg·V/kg/day) for the title compound.

## Figures and Tables

**Table 1:** Induction of the dyslipidemia and dysglycemia model.

	15 days fitting-out	1 month		2 months	
	5001 (*n*=40)	5001 (*n*=10)	5008 (*n*=30)	5001 (*n*=10)	5008 (*n*=30)
Weight (g)	158 ± 6.3	230 ± 7.0	270 ± 5.0 ▲	323 ± 8.7	388 ± 11.4 ▲
Height (cm)	15.3 ± 0.6	20.3 ± 1.7	21.4 ± 1.4	23.2 ± 0.8	24.2 ± 1.1
Abdominal perimeter (cm)	13.9 ± 0.4	15.4 ± 0.6	16.8 ± 0.9	17.6 ± 0.7	18.9 ± 1.1
BMI (g/cm^2^)	0.68 ± 0.07	0.57 ± 0.08	0.59 ± 0.08	0.59 ± 0.03	0.66 ± 0.04
% body fat (Lee index)	34.7 ± 0.9	29.8 ± 2.2	29.8 ± 1.8	29.2 ± 0.9	29.6 ± 1.1
Glucose (mg/dL)	106.0 ± 4.1	104.9 ± 4.5	174.3 ± 3.6 ▲	104.1 ± 6.8	206.3 ± 4.5 ▲
Fructosamine (mmol/L)	40.8 ± 0.8	42.8 ± 1.3	55.3 ± 2.1 ▲	43.2 ± 0.5	60.3 ± 2.3 ▲
Triglycerides (mg/dL)	58.4 ± 3.4	75.0 ± 1.5	104.7 ± 6.7 ▲	70.0 ± 8.2	119.6 ± 5.5 ▲
Total cholesterol (mg/dL)	86.8 ± 1.9	75.2 ± 1.9	72.5 ± 1.4	78.0 ± 2.3	80.0 ± 2.5
HDL (mg/dL)	45.7 ± 1.2	49.1 ± 1.7	41.3 ± 1.0 ▼	45.1 ± 2.0	38.5 ± 1.0 ▼
Insulin (*μ*UI/mL)	15.3 ± 0.5	13.3 ± 0.8	25.9 ± 2.1 ▲	15.5 ± 1.1	33.8 ± 4.2 ▲
HOMA-IR	0.67 ± 0.04	0.58 ± 0.06	1.86 ± 0.19 ▲	0.67 ± 0.09	2.88 ± 0.42 ▲
IDA-IR	0.11 ± 0.01	0.18 ± 0.01	0.4 ± 0.02 ▲	0.19 ± 0.03	0.49 ± 0.01 ▲

Results shown are the average ± SEM. ▲ indicates a significant difference with values above the control group with a normal calorie diet. ▼ indicates a significant difference with values below the control group with a normal calorie diet. Comparisons between groups were performed by Student's *t*-test. BMI = body mass index; HDL = high density lipoprotein; HOMA-IR = homeostasis model assessment insulin resistance; IDA-IR = insulin resistance adipocyte dysfunction.

**Table 2:** Effect of V10-DMAP on biochemical parameters.

	NC (*n*=10)	HC (*n*=10)	HC-V10-DMAP-5 *μ*mol (*n*=10)	HC-V10-DMAP-10 *μ*mol (*n*=10)
Glucose (mg/dL)	112.5 ± 3.5	142.3 ± 6.9 ▲	84.3 ± 2.1 ▼ ↓	94.8 ± 5.4 ▼ ↓
Fructosamine (mmol/L)	44.6 ± 0.8	63.8 ± 1.3 ▲	45.5 ± 2.1 ↓	38.2 ± 1.7 ▼ ↓
Triglycerides (mg/dL)	56.2 ± 3.4	140.0 ± 10 ▲	64.1 ± 2.8 ▲ ↓	60.0 ± 3.2 ↓
FFA (mg/dL)	2.0 ± 0.3	7.4 ± 1.1 ▲	2.2 ± 0.1 ↓	2.0 ± 0.1 ↓
Total cholesterol (mg/dL)	76.9 ± 4.3	105 ± 5.1 ▲	75.2 ± 3.1 ↓	79.0 ± 4.2 ↓
HDL (mg/dL)	43.3 ± 0.9	32.5 ± 1.1 ▼	51.1 ± 1.2 ▲ ↑	48.3 ± 1.5 ▲ ↑
Insulin (*μ*UI/mL)	17.5 ± 0.8	63.4 ± 1.5 ▲	15.2 ± 1.5 ↓	20.2 ± 3.1 ↓
Leptin (ng/mL)	1.1 ± 0.02	5.7 ± 0.2 ▲	1.2 ± 0.09 ↓	2.1 ± 0.1 ↓
Adiponectin (*μ*g/mL)	5.52 ± 0.3	3.21 ± 0.2 ▼	5.12 ± 0.2 ↑	4.32 ± 0.3 ▼ ↑
HOMA-IR	0.81 ± 0.06	3.84 ± 0.14 ▲	0.53 ± 0.06 ▼ ↓	0.81 ± 0.17 ↓
IDA-IR	0.11 ± 0.02	0.63 ± 0.02 ▲	0.09 ± 0.01 ▼ ↓	0.09 ± 0.01 ▼ ↓

Results shown are the average ± SEM. ▲ indicates a significant difference with values above the control group with a normal calorie diet. ▼ indicates a significant difference with values below the control group with a normal calorie diet. ↑ indicates a significant difference with values above the HC group. ↓ indicates a significant difference with values below the HC group. Comparisons between groups were performed by a two-way ANOVA and the Bonferroni post hoc test; *p* < 0.05. FFA = free fatty acids; HDL = high density lipoprotein; HOMA-IR = homeostasis model assessment insulin resistance; IDA-IR = insulin resistance adipocyte dysfunction.

**Table 3:** Toxicological effect of V10-DMAP administration.

	NC (*n*=10)	HC (*n*=10)	HC-V10-DMAP-5 *μ*mol (*n*=10)	HC-V10-DMAP-10 *μ*mol (*n*=10)
Total bilirubin (mg/dL)	0.65 ± 0.03	0.68 ± 0.02	0.62 ± 0.03	0.63 ± 0.04
AST (U/L)	117 ± 8	193 ± 11 ▲	105 ± 6 ↓	148 ± 7 ▲ ↓
ALT (U/L)	65 ± 4	90 ± 7 ▲	59 ± 4 ↓	78 ± 4 ↓
*γ*-GT (U/L)	55 ± 8	76 ± 6 ▲	63 ± 4 ↓	70 ± 5 ▲
LDH (U/L)	200 ± 12	455 ± 15 ▲	220 ± 14 ↓	316 ± 15 ▲ ↓

Results shown are the average ± SEM. ▲ indicates a significant difference with values above the control group with a normal calorie diet. ↓ indicates a significant difference with values below the HC group. Comparisons between groups were performed by a two-way ANOVA and the Bonferroni post hoc test; *p* < 0.05. AST = aspartate aminotransferase; ALT = alanine aminotransferase; *γ*-GT = gamma-glutamyl transpeptidase; LDH = lactate dehydrogenase.

## Data Availability

The data that support the findings of this study are available from the corresponding author upon reasonable request.

## Supplementary Materials

Supplementary 1 Figure S6: kidney histology after V10-DMAP administration.

Supplementary 2 Table S4: renal function after V10-DMAP administration.
