# Supplementary material for: Pharmacological and Toxicological Threshold of Bisammonium Tetrakis 4-(N,N-Dimethylamino)pyridinium Decavanadate in a Rat Model of Metabolic Syndrome and Insulin Resistance
Source: Bioinorg Chem Appl. 2018 Jun 19;2018:2151079. doi: 10.1155/2018/2151079 (PMC6031092; doi:10.1155/2018/2151079)

**Figure S6.** **Kidney Histology after V10-DMAP administration**. A) NC group; B) HC group; C) V10-DMAP-5µmol group; D) V10-DMAP-10µmol group. (*) Bowman’s space, (▼) Tubular lumen, (**→**) endocapillary proliferative glomerulonephritis, ( ) Tubular lumen damage. After cut-off time, the kidney was removed from each animal and perfused with 4% paraformaldehyde (pH 7.4), finally, the tissue was stored in neutral buffered 4% formalin for fixation. Then, tissues were embedded in paraffin and sectioned to a thickness of 5µm, and stained with hematoxylin and eosin. H&E stained sections were viewed under standard bright field illumination. Digital images of representative sections were captured with a Leica microscope and digital camera using the Image-J software. The magnification images were acquired in 40X objective.


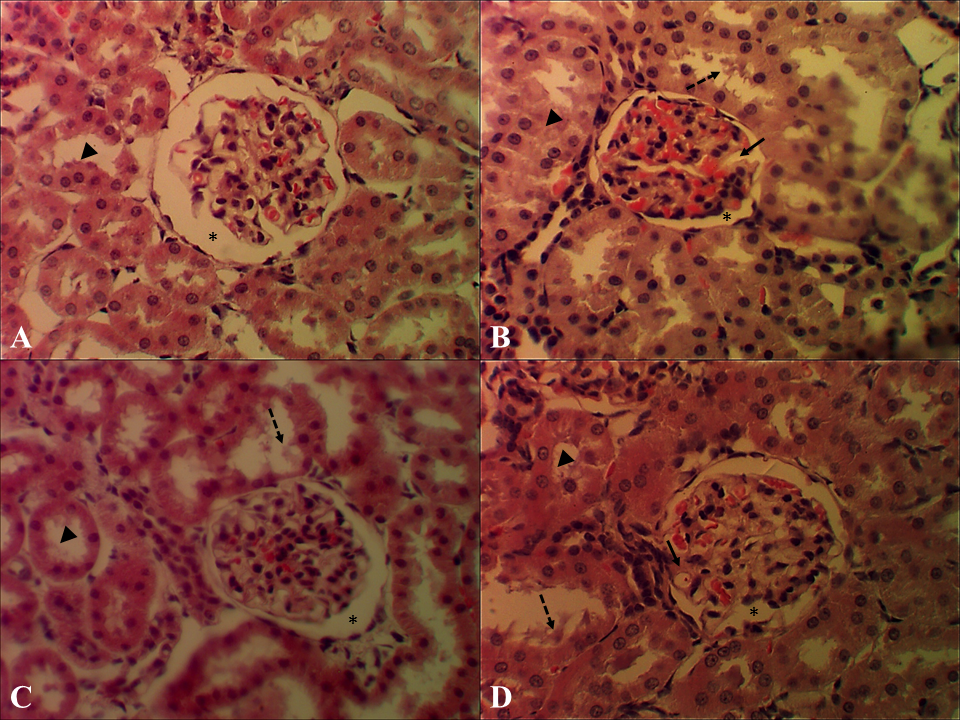

Supplement: Supplementary 1 — Figure S6: kidney histology after V10-DMAP administration. [file 2151079.f1.docx]
