# Supplementary material for: Pharmacological and Toxicological Threshold of Bisammonium Tetrakis 4-(N,N-Dimethylamino)pyridinium Decavanadate in a Rat Model of Metabolic Syndrome and Insulin Resistance
Source: Bioinorg Chem Appl. 2018 Jun 19;2018:2151079. doi: 10.1155/2018/2151079 (PMC6031092; doi:10.1155/2018/2151079)
Supplement: Supplementary 2 — Table S4: renal function after V10-DMAP administration. [file 2151079.f2.docx]

**Table S4. RENAL FUNCTION AFTER V10-DMAP ADMINISTRATION**

|  | **NC**  **(n = 10)** | **HC**  **(n = 10)** | **HC-V10-DMAP-5μmol**  **(n = 10)** | **HC-V10-DMAP-10μmol**  **(n = 10)** |
| --- | --- | --- | --- | --- |
| **Creatinine serum (mg/dL)** | 0.82 ± 0.02 | 2.36 ± 0.08▲ | 0.92 ± 0.06 ↓ | 1.13 ± 0.11 ▲↓ |
| **Urea serum (mg/dL)** | 24 ± 1.1 | 73 ± 3.4 ▲ | 32 ± 8 ↓ | 49 ± 3.4 ▲↓ |
| **Uric Acid serum (mg/dL)** | 2.54 ± 0.4 | 5.31 ± 0.3 ▲ | 3.17 ± 0.7 ↓ | 3.25 ± 0.3 ↓ |
| **eGFR (mL/min)** | 2.0 ± 0.2 | 0.9 ± 0.08▼ | 1.85± 0.15 ↑ | 1.65± 0.23 ↑ |
| **Alb/Cr (mg/g)** | 3.2 ± 0.4 | 45.7 ± 3.4▲ | 4.4 ± 0.6 ↓ | 7.1 ± 1.3 ▲↓ |

Results shown are the average ± SEM. (▲) indicates a significant difference with values above the control group with a normal calorie diet. (▼) indicates a significant difference with values below the control group with a normal calorie diet. (↓) Indicates a significant difference with values below the HC group. (↑) Indicates a significant difference with values above the HC group. Comparisons between groups were performed by a two-way ANOVA and Bonferroni post hoc test; p < 0.05. Urine samples analyzed were 24h-collected for **eGFR (**Estimated Glomerular Filtration Rate) and **Alb/Cr (**Albumin and creatinine ratio).
